# Supplementary material for: Effects of insulin resistance and β-cell function on diabetic complications in Korean diabetic patients
Source: PLoS One. 2024 Oct 22;19(10):e0312439. doi: 10.1371/journal.pone.0312439 (PMC11495573; doi:10.1371/journal.pone.0312439)
Supplement: S1 Table — Data are presented as mean ± standard deviation or as frequency and proportion. HOMA-IR, homeostasis model assessment of insulin resistance; BMI, body mass index; TC, total cholesterol; TG, triglycerides; HDL-C, high-density lipoprotein cholesterol; LDL-C, low-density lipoprotein cholesterol; HbA1c, glycated hemoglobin; HOMA-β, homeostasis model assessment of beta cell function; SBP, systolic blood pressure; DBP, diastolic blood pressure. (DOCX) [file pone.0312439.s001.docx]

S1 Table. Baseline clinical characteristics of participants according to HOMA-IR quartiles

|  | HOMA-IR quartiles | | | | *P* for trend |
| --- | --- | --- | --- | --- | --- |
|  | 1 (n = 508) | 2 (n = 509) | 3 (n = 509) | 4 (n = 508) |  |
| Age (years) | 52 ± 16 | 52 ± 14 | 54 ± 15 | 49 ± 19 | < 0.001 |
| Women, n (%) | 265 (52.2) | 256 (50.3) | 252 (49.5) | 253 (49.8) | 0.833 |
| BMI (kg/m^2^) | 24 ± 3 | 26 ± 4 | 27 ± 4 | 28 ± 5 | < 0.001 |
| TC (mg/dL) | 177 ± 40 | 181 ± 39 | 184 ± 42 | 188 ± 45 | 0.001 |
| TG (mg/dL) | 107 ± 74 | 144 ± 96 | 166 ± 117 | 196 ± 191 | < 0.001 |
| HDL-C (mg/dL) | 53 ± 14 | 49 ± 12 | 47 ± 11 | 46 ± 11 | < 0.001 |
| LDL-C (mg/dL) | 106 ± 33 | 108 ± 35 | 110 ± 35 | 115 ± 37 | 0.001 |
| HbA1c (%) | 6.9 ± 1.5 | 7.3 ± 1.6 | 7.6 ± 1.9 | 7.7 ± 1.9 | < 0.001 |
| Fasting plasma glucose (mg/dL) | 113 ± 32 | 129 ± 40 | 144 ± 46 | 170 ± 62 | < 0.001 |
| Fasting plasma insulin (mIU/L) | 4.0 ± 1.6 | 7.6 ± 2.2 | 11.7 ± 3.4 | 32.8 ± 34.9 | < 0.001 |
| C-peptide (mIU/L) | 1.8 ± 0.9 | 2.4 ± 0.8 | 2.9 ± 1.0 | 4.9 ± 2.8 | < 0.001 |
| HOMA-IR | 1.1 ± 0.4 | 2.3 ± 0.3 | 3.9 ± 0.7 | 13.1 ± 14.0 | < 0.001 |
| HOMA-β (%) | 43.0 ± 38.4 | 65.6 ± 64.2 | 77.2 ± 70.9 | 168.2 ± 229.1 | < 0.001 |
| SBP (mmHg) | 134 ± 21 | 136 ± 22 | 139 ± 22 | 138 ± 21 | 0.218 |
| DBP (mmHg) | 78 ± 12 | 80 ± 11 | 82 ± 15 | 80 ± 14 | 0.169 |
| Hypoglycemic agents, any (%) | 232 (45.7) | 242 (47.5) | 227 (44.6) | 160 (31.5) | < 0.001 |
| Lipid-lowering therapy, any (%) | 139 (27.4) | 164 (32.2) | 156 (30.7) | 107 (21.1) | < 0.001 |
| Antihypertensive agents, any (%) | 143 (28.2) | 135 (26.5) | 155 (30.5) | 124 (24.4) | 0.171 |

Data are presented as mean ± standard deviation or as frequency and proportion.

HOMA-IR, homeostasis model assessment of insulin resistance; BMI, body mass index; TC, total cholesterol; TG, triglycerides; HDL-C, high-density lipoprotein cholesterol; LDL-C, low-density lipoprotein cholesterol; HbA1c, glycated hemoglobin; HOMA-β, homeostasis model assessment of beta cell function; SBP, systolic blood pressure; DBP, diastolic blood pressure.
