# Supplementary material for: Effects of insulin resistance and β-cell function on diabetic complications in Korean diabetic patients
Source: PLoS One. 2024 Oct 22;19(10):e0312439. doi: 10.1371/journal.pone.0312439 (PMC11495573; doi:10.1371/journal.pone.0312439)
Supplement: S2 Table — Data are presented as mean ± standard deviation or as frequency and proportion. HOMA-β, homeostasis model assessment of beta cell function; BMI, body mass index; TC, total cholesterol; TG, triglycerides; HDL-C, high-density lipoprotein cholesterol; LDL-C, low-density lipoprotein cholesterol; HbA1c, glycated hemoglobin; HOMA-IR, homeostasis model assessment of insulin resistance; SBP, systolic blood pressure; DBP, diastolic blood pressure. (DOCX) [file pone.0312439.s002.docx]

S2 Table. Baseline clinical characteristics of participants according to HOMA-β quartiles

|  | HOMA-β | | | | *P* for trend |
| --- | --- | --- | --- | --- | --- |
|  | 1 (n = 508) | 2 (n = 509) | 3 (n = 509) | 4 (n = 508) |  |
| Age (years) | 54 ± 14 | 54 ± 14 | 54 ± 15 | 45 ± 20 | < 0.001 |
| Women, n (%) | 203 (40.0) | 228 (44.8) | 297 (58.4) | 298 (58.7) | < 0.001 |
| BMI (kg/m^2^) | 23.7 ± 3.3 | 25.5 ± 3.5 | 26.4 ± 3.9 | 28.1 ± 5.1 | < 0.001 |
| TC (mg/dL) | 188 ± 45 | 182 ± 41 | 178 ± 40 | 182 ± 39 | 0.004 |
| TG (mg/dL) | 143 ± 138 | 158 ± 149 | 153 ± 110 | 157 ± 124 | 0.222 |
| HDL-C (mg/dL) | 50 ± 13 | 48 ± 12 | 49 ± 12 | 47 ± 12 | 0.005 |
| LDL-C (mg/dL) | 111 ± 36 | 108 ± 35 | 106 ± 34 | 113 ± 34 | 0.003 |
| HbA1c (%) | 8.5 ± 2.3 | 7.4 ± 1.4 | 7.0 ± 1.2 | 6.4 ± 1.1 | < 0.001 |
| Fasting plasma glucose (mg/dL) | 174 ± 61 | 144 ± 49 | 126 ± 33 | 113 ± 33 | < 0.001 |
| Fasting plasma insulin (mIU/L) | 5.1 ± 3.0 | 9.1 ± 5.6 | 12.5 ± 7.1 | 29.4 ± 36.0 | < 0.001 |
| C-peptide (mIU/L) | 2.1 ± 1.1 | 2.7 ± 1.3 | 3.2 ± 1.3 | 4.8 ± 3.2 | < 0.001 |
| HOMA-IR | 2.4 ± 2.3 | 3.9 ± 4.6 | 4.4 ± 4.5 | 9.5 ± 14.5 | < 0.001 |
| HOMA-β (%) | 18.2 ± 7.3 | 40.5 ± 6.8 | 72.3 ± 13.4 | 223.2 ± 215.0 | < 0.001 |
| SBP (mmHg) | 137 ± 21 | 139 ± 25 | 136 ± 20 | 136 ± 20 | 0.612 |
| DBP (mmHg) | 80 ± 12 | 83 ± 13 | 79 ± 14 | 80 ± 14 | 0.069 |
| Hypoglycemic agents, any (%) | 226 (44.5) | 240 (47.2) | 228 (44.8) | 167 (32.9) | < 0.001 |
| Lipid-lowering therapy, any (%) | 138 (27.2) | 157 (30.8) | 161 (31.6) | 110 (21.7) | 0.001 |
| Antihypertensive agents, any (%) | 132 (26.0) | 142 (27.9) | 166 (32.6) | 117 (23.0) | 0.006 |

Data are presented as mean ± standard deviation or as frequency and proportion.

HOMA-β, homeostasis model assessment of beta cell function; BMI, body mass index; TC, total cholesterol; TG, triglycerides; HDL-C, high-density lipoprotein cholesterol; LDL-C, low-density lipoprotein cholesterol; HbA1c, glycated hemoglobin; HOMA-IR, homeostasis model assessment of insulin resistance; SBP, systolic blood pressure; DBP, diastolic blood pressure.
