# Supplementary material for: Effects of insulin resistance and β-cell function on diabetic complications in Korean diabetic patients
Source: PLoS One. 2024 Oct 22;19(10):e0312439. doi: 10.1371/journal.pone.0312439 (PMC11495573; doi:10.1371/journal.pone.0312439)
Supplement: S3 Table — Hazard ratios were adjusted for age, gender, body mass index, and prescriptions for antidiabetic, antihypertensive, and lipid-lowering therapies. HOMA-IR, homeostasis model assessment of insulin resistance; HR, hazard ratio; CI, confidence interval. (DOCX) [file pone.0312439.s003.docx]

S3 Table. Hazard ratios for diabetic nephropathy, diabetic retinopathy, or cardiovascular events according to HOMA-IR quartiles, excluding subjects with C-peptide below 0.6 mIU/L

|  | HOMA-IR quartiles | HR | 95% CI | *P*-value |
| --- | --- | --- | --- | --- |
| Diabetic nephropathy | 1 |  |  |  |
|  | 2 | 1.14 | 0.79–1.65 | 0.476 |
|  | 3 | 1.13 | 0.78–1.62 | 0.529 |
|  | 4 | 1.27 | 0.87–1.85 | 0.215 |
| Diabetic retinopathy | 1 |  |  |  |
|  | 2 | 1.20 | 0.72–2.00 | 0.493 |
|  | 3 | 1.10 | 0.63–1.90 | 0.742 |
|  | 4 | 0.80 | 0.41–1.56 | 0.505 |
| Cardiovascular disease | 1 |  |  |  |
|  | 2 | 1.09 | 0.70–1.70 | 0.691 |
|  | 3 | 1.56 | 1.04–2.35 | 0.034 |
|  | 4 | 1.80 | 1.19–2.72 | 0.005 |
| Coronary events | 1 |  |  |  |
|  | 2 | 0.72 | 0.37–1.43 | 0.349 |
|  | 3 | 1.26 | 0.69–2.29 | 0.459 |
|  | 4 | 1.28 | 0.68–2.39 | 0.446 |
| Cerebrovascular events | 1 |  |  |  |
|  | 2 | 1.38 | 0.80–2.40 | 0.247 |
|  | 3 | 1.62 | 0.96–2.73 | 0.070 |
|  | 4 | 1.99 | 1.19–3.31 | 0.009 |

Hazard ratios were adjusted for age, gender, body mass index, and prescriptions for antidiabetic, antihypertensive, and lipid-lowering therapies.

HOMA-IR, homeostasis model assessment of insulin resistance; HR, hazard ratio; CI, confidence interval.
