# Supplementary material for: Effects of insulin resistance and β-cell function on diabetic complications in Korean diabetic patients
Source: PLoS One. 2024 Oct 22;19(10):e0312439. doi: 10.1371/journal.pone.0312439 (PMC11495573; doi:10.1371/journal.pone.0312439)
Supplement: S4 Table — Hazard ratios were adjusted for age, gender, body mass index, and prescriptions for antidiabetic, antihypertensive, and lipid-lowering therapies. HOMA-β, homeostasis model assessment of beta cell function; HR, hazard ratio; CI, confidence interval. (DOCX) [file pone.0312439.s004.docx]

S4 Table. Hazard ratios for diabetic nephropathy, diabetic retinopathy, or cardiovascular events according to HOMA-β quartiles, excluding subjects with C-peptide below 0.6 mIU/L

|  | HOMA-β quartiles | HR | 95% CI | *P*-value |
| --- | --- | --- | --- | --- |
| Diabetic nephropathy | 1 | 1.08 | 0.72–1.62 | 0.720 |
|  | 2 | 1.17 | 0.80–1.73 | 0.421 |
|  | 3 | 1.11 | 0.75–1.63 | 0.609 |
|  | 4 |  |  |  |
| Diabetic retinopathy | 1 | 3.29 | 1.46–7.41 | 0.004 |
|  | 2 | 1.82 | 0.78–4.24 | 0.166 |
|  | 3 | 1.86 | 0.80–4.36 | 0.151 |
|  | 4 |  |  |  |
| Cardiovascular disease | 1 | 0.60 | 0.39–0.93 | 0.021 |
|  | 2 | 0.77 | 0.51–1.16 | 0.213 |
|  | 3 | 0.77 | 0.50–1.18 | 0.230 |
|  | 4 |  |  |  |
| Coronary events | 1 | 0.61 | 0.33–1.14 | 0.122 |
|  | 2 | 0.53 | 0.28–1.00 | 0.049 |
|  | 3 | 0.60 | 0.31–1.14 | 0.116 |
|  | 4 |  |  |  |
| Cerebrovascular events | 1 | 0.58 | 0.34–1.01 | 0.054 |
|  | 2 | 0.93 | 0.57–1.52 | 0.764 |
|  | 3 | 0.82 | 0.49–1.39 | 0.465 |
|  | 4 |  |  |  |

Hazard ratios were adjusted for age, gender, body mass index, and prescriptions for antidiabetic, antihypertensive, and lipid-lowering therapies.

HOMA-β, homeostasis model assessment of beta cell function; HR, hazard ratio; CI, confidence interval.
