# Supplementary material for: Effects of insulin resistance and β-cell function on diabetic complications in Korean diabetic patients
Source: PLoS One. 2024 Oct 22;19(10):e0312439. doi: 10.1371/journal.pone.0312439 (PMC11495573; doi:10.1371/journal.pone.0312439)
Supplement: S5 Table — Hazard ratios were adjusted for age, gender, body mass index, and prescriptions for antidiabetic, antihypertensive, and lipid-lowering therapies. HOMA-IR, homeostasis model assessment of insulin resistance; HR, hazard ratio; CI, confidence interval. (DOCX) [file pone.0312439.s005.docx]

S5 Table. Hazard ratios for diabetic nephropathy, diabetic retinopathy, or cardiovascular events according to HOMA-IR quartiles with the follow-up period is less than 2 years

|  | HOMA-IR quartiles | HR | 95% CI | *P*-value |
| --- | --- | --- | --- | --- |
| Diabetic nephropathy | 1 |  |  |  |
|  | 2 | 1.18 | 0.81-1.71 | 0.381 |
|  | 3 | 1.15 | 0.80-1.67 | 0.451 |
|  | 4 | 1.25 | 0.86-1.83 | 0.248 |
| Diabetic retinopathy | 1 |  |  |  |
|  | 2 | 0.92 | 0.35-2.40 | 0.864 |
|  | 3 | 0.60 | 0.20-1.84 | 0.373 |
|  | 4 | 0.76 | 0.24-2.39 | 0.633 |
| Cardiovascular disease | 1 |  |  |  |
|  | 2 | 1.70 | 1.03-2.79 | 0.038 |
|  | 3 | 2.10 | 1.31-3.37 | 0.002 |
|  | 4 | 2.08 | 1.30-3.34 | 0.002 |
| Coronary events | 1 |  |  |  |
|  | 2 | 1.27 | 0.55-2.92 | 0.578 |
|  | 3 | 2.11 | 1.01-4.42 | 0.048 |
|  | 4 | 1.92 | 0.90-4.09 | 0.093 |
| Cerebrovascular events | 1 |  |  |  |
|  | 2 | 2.08 | 1.14-3.80 | 0.017 |
|  | 3 | 2.04 | 1.13-3.68 | 0.018 |
|  | 4 | 2.01 | 1.12-3.61 | 0.019 |

Hazard ratios were adjusted for age, gender, body mass index, and prescriptions for antidiabetic, antihypertensive, and lipid-lowering therapies.

HOMA-IR, homeostasis model assessment of insulin resistance; HR, hazard ratio; CI, confidence interval.
