# Supplementary material for: Effects of insulin resistance and β-cell function on diabetic complications in Korean diabetic patients
Source: PLoS One. 2024 Oct 22;19(10):e0312439. doi: 10.1371/journal.pone.0312439 (PMC11495573; doi:10.1371/journal.pone.0312439)
Supplement: S6 Table — Hazard ratios were adjusted for age, gender, body mass index, and prescriptions for antidiabetic, antihypertensive, and lipid-lowering therapies. HOMA-β, homeostasis model assessment of beta cell function; HR, hazard ratio; CI, confidence interval. (DOCX) [file pone.0312439.s006.docx]

S6 Table. Hazard ratios for diabetic nephropathy, diabetic retinopathy, or cardiovascular events according to HOMA-β quartiles with the follow-up period is less than 2 years

|  | HOMA-β quartiles | HR | 95% CI | *P*-value |
| --- | --- | --- | --- | --- |
| Diabetic nephropathy | 1 | 1.25 | 0.83-1.89 | 0.291 |
|  | 2 | 1.28 | 0.87-1.90 | 0.209 |
|  | 3 | 1.26 | 0.86-1.84 | 0.241 |
|  | 4 |  |  |  |
| Diabetic retinopathy | 1 | 6.28 | 0.77-51.27 | 0.086 |
|  | 2 | 4.96 | 0.61-40.47 | 0.135 |
|  | 3 | 5.92 | 0.74-47.68 | 0.095 |
|  | 4 |  |  |  |
| Cardiovascular disease | 1 | 1.00 | 0.61-1.64 | 1.000 |
|  | 2 | 1.36 | 0.86-2.16 | 0.185 |
|  | 3 | 1.02 | 0.63-1.65 | 0.947 |
|  | 4 |  |  |  |
| Coronary events | 1 | 1.23 | 0.58-2.62 | 0.591 |
|  | 2 | 0.81 | 0.36-1.81 | 0.609 |
|  | 3 | 1.02 | 0.48-2.19 | 0.962 |
|  | 4 |  |  |  |
| Cerebrovascular events | 1 | 0.82 | 0.44-1.51 | 0.521 |
|  | 2 | 1.54 | 0.90-2.64 | 0.118 |
|  | 3 | 0.96 | 0.53-1.71 | 0.880 |
|  | 4 |  |  |  |

Hazard ratios were adjusted for age, gender, body mass index, and prescriptions for antidiabetic, antihypertensive, and lipid-lowering therapies.

HOMA-β, homeostasis model assessment of beta cell function; HR, hazard ratio; CI, confidence interval.
