# Supplementary material for: Effects of insulin resistance and β-cell function on diabetic complications in Korean diabetic patients
Source: PLoS One. 2024 Oct 22;19(10):e0312439. doi: 10.1371/journal.pone.0312439 (PMC11495573; doi:10.1371/journal.pone.0312439)
Supplement: Attachment — Submitted filename: Response to Reviewers.docx [file pone.0312439.s007.docx]

Journal Requirements:

When submitting your revision, we need you to address these additional requirements.

1. Please ensure that your manuscript meets PLOS ONE's style requirements, including those for file naming. The PLOS ONE style templates can be found at <https://journals.plos.org/plosone/s/file?id=wjVg/PLOSOne_formatting_sample_main_body.pdf> and  <https://journals.plos.org/plosone/s/file?id=ba62/PLOSOne_formatting_sample_title_authors_affiliations.pdf>

→ Thanks for your comment. We revised the manuscript to conform to PLOS ONE's style requirements.

2. Thank you for stating the following in your Competing Interests section:  
"No potential conflict of interest relevant to this article was reported."

Please complete your Competing Interests on the online submission form to state any Competing Interests. If you have no competing interests, please state "The authors have declared that no competing interests exist.", as detailed online in our guide for authors at http://journals.plos.org/plosone/s/submit-now 
 This information should be included in your cover letter; we will change the online submission form on your behalf.

→ Thank you for your consideration. "The authors have declared that no competing interests exist."

3. We note that your Data Availability Statement is currently as follows: [All relevant data are within the manuscript and its Supporting Information files.]

Please confirm at this time whether or not your submission contains all raw data required to replicate the results of your study. Authors must share the “minimal data set” for their submission. PLOS defines the minimal data set to consist of the data required to replicate all study findings reported in the article, as well as related metadata and methods (https://journals.plos.org/plosone/s/data-availability#loc-minimal-data-set-definition).

For example, authors should submit the following data:

- The values behind the means, standard deviations and other measures reported;
- The values used to build graphs;
- The points extracted from images for analysis.

Authors do not need to submit their entire data set if only a portion of the data was used in the reported study.

If your submission does not contain these data, please either upload them as Supporting Information files or deposit them to a stable, public repository and provide us with the relevant URLs, DOIs, or accession numbers. For a list of recommended repositories, please see <https://journals.plos.org/plosone/s/recommended-repositories>.

→ Thanks for your comment. Our submission contains all raw data required to replicate the results of our study.

If there are ethical or legal restrictions on sharing a de-identified data set, please explain them in detail (e.g., data contain potentially sensitive information, data are owned by a third-party organization, etc.) and who has imposed them (e.g., an ethics committee). Please also provide contact information for a data access committee, ethics committee, or other institutional body to which data requests may be sent. If data are owned by a third party, please indicate how others may request data access.

[Note: HTML markup is below. Please do not edit.]

Reviewers' comments:

Reviewer's Responses to Questions

**Comments to the Author**

1. Is the manuscript technically sound, and do the data support the conclusions?

The manuscript must describe a technically sound piece of scientific research with data that supports the conclusions. Experiments must have been conducted rigorously, with appropriate controls, replication, and sample sizes. The conclusions must be drawn appropriately based on the data presented.

Reviewer #1: Yes

Reviewer #2: Yes

Reviewer #3: Partly

2. Has the statistical analysis been performed appropriately and rigorously?

Reviewer #1: Yes

Reviewer #2: No

Reviewer #3: Yes

3. Have the authors made all data underlying the findings in their manuscript fully available?

The [PLOS Data policy](http://c05y1x9s.r.us-east-2.awstrack.me/L0/http:%2F%2Fwww.plosone.org%2Fstatic%2Fpolicies.action%23sharing/1/010f01915b8df3e0-c15da6cf-35fc-4771-a634-0e6b71e1e8a3-000000/LLlMXXaVfmeNfzOFtan6tCqtd1A=172) requires authors to make all data underlying the findings described in their manuscript fully available without restriction, with rare exception (please refer to the Data Availability Statement in the manuscript PDF file). The data should be provided as part of the manuscript or its supporting information, or deposited to a public repository. For example, in addition to summary statistics, the data points behind means, medians and variance measures should be available. If there are restrictions on publicly sharing data—e.g. participant privacy or use of data from a third party—those must be specified.

Reviewer #1: Yes

Reviewer #2: Yes

Reviewer #3: No

4. Is the manuscript presented in an intelligible fashion and written in standard English?

PLOS ONE does not copyedit accepted manuscripts, so the language in submitted articles must be clear, correct, and unambiguous. Any typographical or grammatical errors should be corrected at revision, so please note any specific errors here.

Reviewer #1: Yes

Reviewer #2: Yes

Reviewer #3: Yes

5. Review Comments to the Author

Please use the space provided to explain your answers to the questions above. You may also include additional comments for the author, including concerns about dual publication, research ethics, or publication ethics. (Please upload your review as an attachment if it exceeds 20,000 characters)

Reviewer #1: In the manuscript titled "Effects of insulin resistance and β-cell function on diabetic complications in Korean diabetic patients," the authors explore how insulin resistance and β-cell function relate to diabetic complications in Korean patients. This research question is compelling, and the findings are well-supported, offering potential clinical significance. With some adjustments for clarity and detail, this work should be ready for publication.
•
The abstract could be improved by including more specifics about the clinical implications of the findings.
→ Thanks for your comment. “Individuals with pronounced IR should prioritize CVD prevention measures, and those with significant β-cell dysfunction may benefit from early, intensive surveillance for diabetic retinopathy.” was inserted in the Abstract (line 40-42).

The introduction does a good job of outlining the complex nature of diabetes. However, it could be clearer about the specific gaps in the current research that this study addresses, especially regarding Korean diabetic patients. It would help to emphasize why it's important to study this particular group.
→ Thanks for your comment. “Severe β-cell dysfunction group had lower odds of chronic kidney disease and severe insulin resistance group had higher odds of carotid artery plaque presence among patients with type 2 diabetes in Korea. However, little research has been conducted on the relationship between IR and β-cell function in relation to multiple diabetic complications among Korean diabetic patients.” were inserted in the Introduction section (line 75-79).

The methods section is detailed, explaining the use of a common data model (CDM) and defining how insulin resistance and β-cell function were measured. It would be useful to include more details about how patients were selected and the specific statistical methods used to calculate hazard ratios. Addressing potential biases and how they were mitigated would strengthen this section. It’s also important to explain how patient data was anonymized and how any missing data was managed.

→ “Among the total subjects, there were 9,107 adults aged 20 years or older for whom HOMA-IR and HOMA-β values could be calculated. Our study population comprised 4,113 subjects aged over 20 years who had been diagnosed with diabetes mellitus and had visited Ewha Womans University Mokdong Hospital between January 1, 2001, and December 31, 2019. We sourced these records from the CDM database.” (line 101-105)

“We excluded subjects with incomplete data (n = 13), those with a history of diabetic nephropathy (n = 139), diabetic retinopathy (n = 104), or CVD (n = 1,615), as well as those who had received insulin prescriptions for more than 90 days from the index date (n = 861). Finally, the study cohort consisted of 2,034 diabetic patients aged over 20 years.” (line 114)

“We calculated HR in three ways: model 1 was unadjusted, model 2 was adjusted for age and gender, and model 3 was adjusted for age, gender, BMI, and medication use, including antidiabetic, antihypertensive, and lipid-lowering therapies.” were inserted in the Methods section (line 148-151).

“Since subjects for whom HOMA-IR and HOMA-β values could not be measured were excluded from the study, selection bias may have affected the study results.” was inserted in the Discussion section (line 298-300).

• For clarity, some sentences could be simplified. For instance, instead of "While approximately 75–85% of diabetic individuals fit the traditional criteria for type 2 diabetes...," you could say, "Approximately 75–85% of diabetic patients meet the traditional criteria for type 2 diabetes, but many show characteristics of both insulin resistance and β-cell dysfunction."
→ Thanks for your comment. As you recommended, we changed the sentences to make it more concise.

The discussion should compare the findings with previous studies, particularly those involving other ethnic groups, to highlight the unique aspects of the results. The conclusion could more clearly outline the practical implications, such as how these findings might influence screening or treatment decisions based on insulin resistance and β-cell function. Discussing potential reasons behind the associations found, such as cultural or genetic factors specific to Korean populations, would add depth.

→ Thanks for your comment.

“A previous study of German patients newly diagnosed with diabetes found that the prevalence of nonalcoholic fatty liver disease and diabetic neuropathy varied across specific diabetes populations.” (line 293-295)

“Because IR was known to be associated with several risk factors for CVD including hyperglycemia, dyslipidemia, and hypertension [9, 10], so in this study, patients with diabetes and increased IR may have an increased risk of CVD. Previous epidemiologic studies have shown that β-cell dysfunction was associated with an increased risk of severe diabetic retinopathy [24-26], but the mechanism is unclear. β-cell dysfunction may be a surrogate marker of diabetes duration and more progressive forms of diabetes.” (line 305-310)

“In order to prevent and manage complications in Korean diabetic patients, it is necessary to understand the pathophysiology of Korean diabetic patients. It is also necessary to identify subgroups vulnerable to diabetic complications and thoroughly screen them in advance to manage complications.” (line 314-317)

“Genetic and environmental factors may affect the development of diabetes, and differences in genetic and environmental factors by race may have affected different results across studies.” was inserted in the Discussion section (line 261-263).

Reviewer #2: Authors looked into the dose-response relationship of beta-cell function and diabetes complications in diabetic individuals.

Abstract is poor. Patient characteristics must be stated in the results section not method. A brief explanation of your data source will be useful in the methods. Also confidence intervals for adjusted hazard ratios must be reported.

→ Thanks for your comment. “The study cohort consisted of 2,034 diabetic patients aged over 20 years who visited EUMC between January 2001 and December 2019.” was stated in the results section. We included confidence intervals for adjusted hazard ratios in the abstract.

Second paragraph of the introduction was too long. I think you can summarize it and talk less about the swedish diabetes classification and more about the importance of beta-cell function and diabetes complications.
→ Thanks for your comment. We summarize second paragraph of the introduction and talk less about the swedish diabetes classification.

Paragraph 3 in introduction: Aim of study is not clear. Why, despite the other similar papers in different countries, it is important to investigate the relationship in Korean population?

→ Thanks for your comment. “Genetic and environmental factors can influence the development of diabetes. Korean diabetic patients are known to have low insulin secretion ability, but as the number of overweight and obese diabetic patients increases, insulin resistance is considered a more prominent pathophysiology of diabetes.” were inserted in the Introduction section (line 71-74).

Your follow-up duration is vague. You did not report it anywhere as I check. Please calculate it. Moreover, I also suggest subgroup analysis on those with a follow-up duration <2 years (Short-term outcome).
→ Thanks for your comment. “with a mean follow-up of two years.” was inserted in the Results section. “In additional sensitivity analysis with the follow-up period is less than 2 years, results were similar (refer to Supplemental Tables S5 and S6). Among diabetic patients in the highest quartile of HOMA-IR, the adjusted hazard ratio for total CVD events was 2.08 (95% CI, 1.30-3.34; *P* = 0.002), and for stroke, it was 2.01 (95% CI, 1.12-3.61; *P* = 0.019) when compared with those in the lowest quartile of HOMA-IR. For diabetic patients in the lowest quartile of HOMA-β, there was no statistically significant difference, but the adjusted hazard ratio for diabetic retinopathy was 6.28 (95% CI, 0.77-51.27; *P* = 0.086) relative to those in the highest quartile of HOMA-β.” were inserted in the Results section (line 207-214).

Did you include all the variables in your regression model for adjustment? Or just the statistically significant variables. I suggest to perform three regression models to have a better comparison: model 1= unadjusted, model 2= age and sex adjusted, model 3= adjusted with all confounding variables.
→ Thanks for your comment. Three regression models are shown in Tables 3 and 4.

Baseline characteristics of the patients must be stated using numbers (at least for important variables such as age, sex,
→ Thanks for your comment. Baseline characteristics of the patients are shown in Tables 1 and 2. “Fifty percent (n = 1,008) of the total subjects were male and the mean age was 51.6 years.” was inserted in the Results section (line 159-160).

Results were poorly organized. I think separation into sections based on different diabetic complications such as retinopathy, nephropathy, and CVD, and then report each relevant finding in its specific section might be suitable.
→ Thanks for your comment. We divided the sections based on different diabetic complications such as retinopathy, nephropathy, and CVD.

Again all the confidence intervals are missed. Please report. Also provide p-values and statistical tests for tables 1&2.
→ Thanks for your comment. We revised the results by adding confidence intervals. And we provided *p*-values and statistical tests for Tables 1 and 2.

Discussion is well-written. However, adding a paragraph about the possible molecular mechanisms underneath the association of diabetic retinopathy or CVD risk with beta-cell function and insulin resistance.

→ Thanks for your comment. “Because IR was known to be associated with several risk factors for CVD including hyperglycemia, dyslipidemia, and hypertension [9, 10], so in this study, patients with diabetes and increased IR may have an increased risk of CVD. Previous epidemiologic studies have shown that β-cell dysfunction was associated with an increased risk of severe diabetic retinopathy, but the mechanism is unclear. β-cell dysfunction may be a surrogate marker of diabetes duration and more progressive forms of diabetes.” were inserted in the Discussion section (line 304-310).

Reviewer #3: Review Comment:
1- The study investigates the associations between insulin resistance (IR), β-cell function, and diabetic complications using a cohort from a common data model (CDM) database. While the topic is relevant, the study's novelty is not well articulated in the abstract, which raises concerns about its contribution to the existing body of knowledge.
→ Thanks for your comment. “However, little research has been conducted on the relationship between IR and β-cell function in relation to diabetic complications among Korean diabetic patients.” was inserted in the Abstract.

2- Methodology: The study employs appropriate methodologies, including the calculation of HOMA-IR and HOMA-β, and robust statistical analyses using Cox proportional hazards models. The inclusion of anthropometric measurements, laboratory tests, and detailed statistical adjustments for variables such as age, gender, BMI, and medication use strengthens the study’s methodology. However, the lack of demographic details such as age, gender, and race are significant omissions that hinder the assessment of the study’s generalizability. The exclusion criteria, particularly the exclusion of subjects with incomplete data or prior complications, need more justification to ensure that the study's findings are not biased. Additionally, the stratification of patients based on quartiles of HOMA-IR and HOMA-β is appropriate, but the rationale behind these specific stratifications and reference categories should be clarified.
→ Thanks for your comment. We excluded subjects with incomplete data (n = 13), those with a history of diabetic nephropathy (n = 139), diabetic retinopathy (n = 104), or CVD (n = 1,615), as well as those who had received insulin prescriptions for more than 90 days from the index date (n = 861). “Since subjects for whom HOMA-IR and HOMA-β values could not be measured were excluded from the study, selection bias may have affected the study results.” was described in the Discussion section as limitations of the study.

“Because HOMA-IR and HOMA-β values were not normally distributed, we divided participants into quartiles of HOMA-IR and HOMA-β values.” was inserted in the Method section (line 143-145). Analysis using quartile values of OGTT measurements is consistent with the analysis methods in other papers [10, 15, 19].”
3- Ethical Considerations: The authors mention that the study received approval from the hosting institute and used anonymized data, which addresses privacy concerns. However, the absence of informed consent is a critical issue. The abstract does not clearly state whether this research qualifies as a split study, where data originally collected for a different purpose are repurposed. It’s important that the authors clarify this point and detail how ethical approval covered the secondary use of such data.
→ Thanks for your comment. We conducted a retrospective analysis using the medical records and test results of the hospital where the patient received treatment previously, so no additional tests were required, and even if the research were conducted without consent, the risk to the subject were extremely low, and obtaining consent were realistically impossible during the research process. Moreover, we conducted the research using CDM data from which personal information had already been deleted, so there was no reason to assume that the research subject would refuse consent.
4- References: The study cites only 15 references, which seems relatively few given the complexity of the topic. This limited reference list may indicate that the study has not fully engaged with the breadth of existing literature, particularly in a field as well-studied as diabetes and its complications. A more comprehensive review of relevant studies would strengthen the contextualization and credibility of the findings.
→ Thanks for your comment. As you recommended, we added references.

5- Conclusion: While the study potentially offers valuable insights, several aspects need further clarification and expansion. The abstract should clearly articulate the novelty of the research, include demographic information, address ethical concerns related to the possible use of previously collected data, and consider expanding the reference list to demonstrate a thorough engagement with the existing literature. These major revisions would significantly enhance the rigor and transparency of the study.

→ Thanks for your comment. We have revised the paper as per your comments.

6. PLOS authors have the option to publish the peer review history of their article ([what does this mean?](https://c05y1x9s.r.us-east-2.awstrack.me/L0/https:%2F%2Fjournals.plos.org%2Fplosone%2Fs%2Feditorial-and-peer-review-process%23loc-peer-review-history/1/010f01915b8df3e0-c15da6cf-35fc-4771-a634-0e6b71e1e8a3-000000/r8dWovVprik7iO7U9SM5qcAv4CU=172)). If published, this will include your full peer review and any attached files.

If you choose “no”, your identity will remain anonymous but your review may still be made public.

**Do you want your identity to be public for this peer review?** For information about this choice, including consent withdrawal, please see our [Privacy Policy](https://c05y1x9s.r.us-east-2.awstrack.me/L0/https:%2F%2Fwww.plos.org%2Fprivacy-policy/1/010f01915b8df3e0-c15da6cf-35fc-4771-a634-0e6b71e1e8a3-000000/Ej7Q0hPyc6i8K5J_Vijp7cFWoxI=172).

Reviewer #1: **Yes:** Solaleh Emamgholipour

Reviewer #2: No

Reviewer #3: No

[NOTE: If reviewer comments were submitted as an attachment file, they will be attached to this email and accessible via the submission site. Please log into your account, locate the manuscript record, and check for the action link "View Attachments". If this link does not appear, there are no attachment files.]

While revising your submission, please upload your figure files to the Preflight Analysis and Conversion Engine (PACE) digital diagnostic tool, [https://pacev2.apexcovantage.com/](https://c05y1x9s.r.us-east-2.awstrack.me/L0/https:%2F%2Fpacev2.apexcovantage.com%2F/1/010f01915b8df3e0-c15da6cf-35fc-4771-a634-0e6b71e1e8a3-000000/elRlZ7Z4lNpiBnejSn-39bOTcdM=172). PACE helps ensure that figures meet PLOS requirements. To use PACE, you must first register as a user. Registration is free. Then, login and navigate to the UPLOAD tab, where you will find detailed instructions on how to use the tool. If you encounter any issues or have any questions when using PACE, please email PLOS at [figures@plos.org](mailto:figures@plos.org). Please note that Supporting Information files do not need this step.
